# Another Curiosity

**Published:** 1898-06

**Authors:** 


### ANOTHER CURIOSITY.

B——, MARCHS 18 1898

DEAR SIR. DR.

I would Kindly ask you to please and send me a pescreption for injecting the disease amongist Cattle Called tuberculous and the quantity to-use? and oblige me

DR. X—— Z——

And still they say there is no need for State examining boards.—[ED.].

